# Mammographic casting-type calcification is an independent prognostic factor in invasive breast cancer

**DOI:** 10.1038/s41598-019-47118-3

**Published:** 2019-07-22

**Authors:** Yan Li, Jian Cao, Yidong Zhou, Feng Mao, Songjie Shen, Qiang Sun

**Affiliations:** 1 Department of Breast Surgery, Peking Union Medical College Hospital, Peking Union Medical College, Chinese Academy of Medical Sciences, Beijing, 100730 P.R. China; 2 Department of Radiology, Peking Union Medical College Hospital, Peking Union Medical College, Chinese Academy of Medical Sciences, Beijing, 100730 P.R. China

**Keywords:** Breast cancer, Cancer imaging

## Abstract

This study aimed to determine whether there is an association between mammographic casting-type calcification and other prognostic factors for invasive breast cancer. We also assessed whether casting-type calcification could be an independent prognostic factor. Invasive breast cancer patient information from January 2010 and January 2013 was retrospectively reviewed. The associations between mammographic casting-type calcification and other clinicopathological factors, including tumor size, node status, grade, progesterone receptor (PR) status, estrogen receptor (ER) status, and human epidermal growth factor receptor 2 (HER2) status, were analyzed. The Kaplan–Meier method and a Cox proportional hazards model were used for survival analyses of disease-free survival (DFS) and overall survival (OS). A total of 1155 invasive breast cancer patients who underwent definitive surgery were included, and 136 cases (11.8%) had casting-type calcification on mammography. In multivariate logistic regression, casting-type calcification was significantly associated with axillary node metastasis, ER-negativity, and HER2 overexpression. Casting-type calcification significantly decreased OS and DFS after a median follow-up of 60 months. This result remained after adjusting other prognostic factors in the multivariate analysis. Casting-type calcification is significantly linked to axillary node metastasis, ER-negativity and HER2 overexpression. Casting-type calcification is therefore an independent prognostic factor for breast cancer patients.

## Introduction

Mammographic imaging is an essential tool in screening for breast cancer and deriving a diagnosis^1^. Calcifications that are detected with mammography can be described according to their morphology, size and distribution^2^. Casting-type calcification, which has a long, fine linear branching structure, is a subtype of mammographically detectable microcalcification, and is highly suggestive of malignancy of breast cancer^3^. However, the prognostic value of casting-type calcification is still controversial. For tumors that measured <15 mm detected by mammography screening, casting-type calcification has been demonstrated to be a prognostic factor and it carries a significantly (9-fold) higher risk of death compared to those without this mammographic abnormality^4^. A study recently reported that the presence of casting-type calcifications was associated with a 3.47-fold increased hazard ratio for mortality, after correcting for other prognostic factors^5^. However, conflicting results have also been published^6,7^. A study that analyzed a large cohort of screening-detected invasive breast cancers showed that the presence of mammographic comedo (casting) calcification did not have an influence on breast-cancer-specific survival^8^. Until now, casting-type calcification has not been established as a prognostic factor in clinical practice.

Other studies have reported that breast tumors with casting-type calcifications had an increased rate of HER2 overexpression and ER negativity^9,10^. Several other studies have also indicated there are increased rates of lymph node involvement and larger tumor sizes in patients with calcifications^11,12^. However, other studies reported inconsistent results^7,13^.

In this study, we examined the association between casting-type calcification and clinicopathological factors. We took into account node status, tumor size, tumor biomarkers, and histological grade. Next, we determined whether casting-type calcification could be employed as a reliable prognostic factor for invasive breast cancer.

## Results

### Patient and tumor characteristic

A total of 1155 invasive breast cancer patients who underwent definitive surgery at Peking Union Medical College Hospital (PUMCH) were included in this study. All participants were female and of Asian descent. At diagnosis, the median age of the patients was 47 years and 21% to 83.31% of the patients underwent breast conserving surgery, while 69% of them underwent mastectomy. 36% of them received sentinel lymph node biopsy, and 64% of them received axillary lymph node dissection. Postoperative adjuvant treatment was planned according to the current National Comprehensive Cancer Network (NCCN) guidelines. A total of 83% of the patients received adjuvant chemotherapy including anthracycline-based or taxane-based regimens. Endocrine therapy and trastuzumab were used according to the pathological biomarkers and patients’ actual situation.

Among the 1155 participants, 136 cases (11.8%) had casting-type calcifications on mammography (casting group), and 1019 cases (88.2%) had no casting-type calcifications (non-casting group) (Table 1). According to a t-test, there was no significant difference in age between the two groups (47.36 vs 46.65, *P* = 0.556). A Chi-squared test analysis revealed that tumor size (*P* = 0.937), menstrual status (*P* = 0.868), and tumor grade (*P* = 0.123) were not significantly different between the casting group and non-casting group. However, axillary node status (*P* < 0.001), PR (*P* = 0.030), ER (*P* = 0.009), and HER2 (*P* < 0.001) status showed significant differences.

**Table 1:** Patient characteristics within subgroups.

Characteristic	Non-casting group (n = 1019)	Casting group (n = 136)	P*
Age, years			0.556
Mean ± SD	47.36 ± 13.46	46.65 ± 11.16	
Menstrual status, No. (%)			0.868
Premenopausal	520 (51.03)	72 (52.94)	
Postmenopausal	429 (42.10)	56 (41.18)	
Unknown	70 (6.87)	8 (5.88)	
Tumor size, No. (%)			0.937
T1	486 (47.69)	63 (46.32)	
T2	400 (39.25)	54 (39.71)	
T3	133 (13.05)	19 (13.97)	
Tumor grade, No. (%)			0.123
G1	235 (23.06)	23 (16.91)	
G2	431 (42.30)	69 (50.74)	
G3	353 (34.64)	44 (32.35)	
Axillary node metastasis, No. (%)			<0.001
No	815 (79.98)	89 (65.44)	
Yes	204 (20.02)	47 (34.56)	
ER, No. (%)			0.009
Positive	636 (62.41)	69 (50.74)	
Negative	383 (37.59)	67 (49.26)	
PR, No. (%)			0.030
Positive	587 (57.61)	65 (47.79)	
Negative	432 (42.39)	71 (52.21)	
HER2, No. (%)			<0.001
Positive	208 (20.41)	54 (39.71)	
Negative	762 (74.78)	75 (55.15)	
Unknown	49 (4.81)	7 (5.18)	

SD, standard deviation; ER, estrogen receptor; PR, progesterone receptor; HER2, human epidermal growth factor receptor 2; *Categorical data were compared using a two-tailed chi-squared test. Quantitative data were compared by Student’s t-test. Differences were considered significant at *P* < 0.05.

Patient histologic grade, tumor size, lymph node status, PR, ER, and HER2 were included in the multivariate logistic regression analysis (Table 2). The results suggested that casting-type calcification was significantly associated with ER-negativity (OR 1.49, axillary node metastasis (OR 1.98, 95% CI 1.32–2.95, *P* = 0.001), 95% CI 1.03–2.16, *P* = 0.035), and HER2 overexpression (OR 2.39, 95% CI 1.61–3.53, *P* < 0.001). There was a tendency toward casting-type calcification occurring more frequently among patients with higher histologic grades (OR 1.62, 95% CI 0.97–2.71, *P* = 0.063, for G2 vs. G1; OR 1.24, 95% CI 0.73–2.15, *P* = 0.422, for G3 vs. G1, respectively) and PR-negative tumors (OR 1.40, 95% CI 0.97–2.03, *P* = 0.071,), although the correlation was not significant.

**Table 2:** Multivariate logistic analysis of association between casting-type calcification and tumor characteristics.

Characteristic	Casting-type calcification, No. (%)	OR	95% CI	*P*
Tumor size				
T1	63 (11.48)	Reference		
T2	54 (11.89)	1.02	0.69–1.52	0.907
T3	19 (12.50)	0.89	0.50–1.57	0.686
Tumor grade				
G1	23 (8.91)	Reference		
G2	69 (13.8)	1.62	0.97–2.71	0.063
G3	44 (11.08)	1.24	0.73–2.15	0.422
Axillary node metastasis				
No	89 (9.85)	Reference		
Yes	47 (18.73)	1.98	1.32–2.95	0.001
ER				
Positive	69 (9.79)	Reference		
Negative	67 (14.89)	1.49	1.03–2.16	0.035
PR				
Positive	65 (9.97)	Reference		
Negative	71 (14.12)	1.40	0.97–2.03	0.071
HER2				
Negative	75 (8.96)	Reference		
Positive	54 (20.61)	2.39	1.61–3.53	<0.001
Unknown	7 (12.50)	1.25	0.54–2.90	0.602

OR, odds ratio; CI, confidence interval.

### Survival analysis between casting group and non-casting group

**Figure 1:**
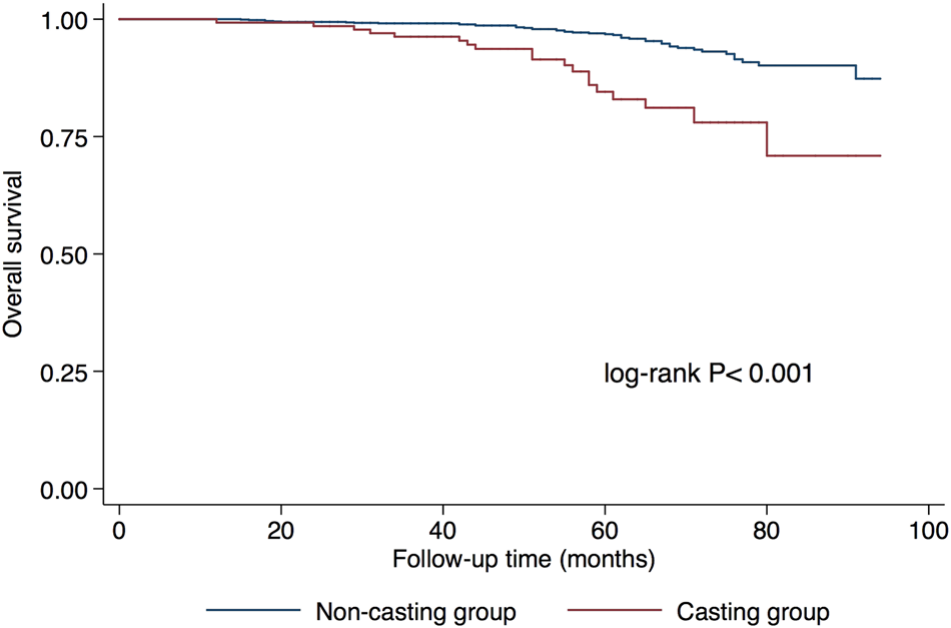
Overall survival curves according to mammographic casting-type calcification. log-rank *P* < 0.001.

The median follow-up time of the analysis was 60 months (range: 12–94). The 5-year Kaplan-Meier estimates for OS were 84.53% in the casting group and 96.80% in the non-casting group (Fig. 1). The log-rank comparison indicated a significant difference in OS (p < 0.001).

**Figure 2:**
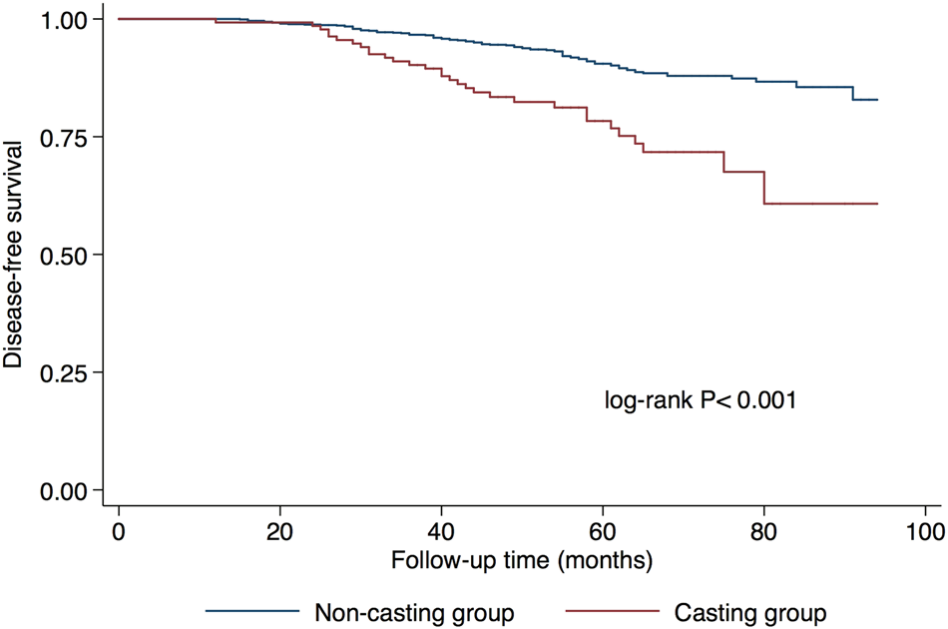
Disease-free survival curves according to mammographic casting-type calcification. log-rank *P* < 0.001.

The 5-year Kaplan-Meier estimates for DFS were 78.34% in the casting group and 90.50% in the non-casting group (Fig. 2). The log-rank comparison also indicated there was a significant difference in DFS (p < 0.001).

### Multivariate analysis for DFS and OS

The included prognostic factors for OS and DFS in the multivariate analysis were tumor grade, tumor size, axillary lymph node status, PR, ER, HER2, and casting-type calcification (Table 3). Casting-type calcification maintained its significance as a prognostic factor for DFS and OS after adjustment for all the other factors (OS HR = 1.95; 95% CI, 1.08–3.53, *P* = 0.026; DFS HR = 1.65; 95% CI, 1.07–2.56, *P* = 0.024).

**Table 3:** Multivariate Cox proportional hazards regression model analysis of disease-free survival and overall survival.

Factor	Disease-free survival			Overall survival		
	HR	95% CI	*P*	HR	95% CI	*P*
Tumor size						
T1	Reference			Reference		
T2	1.62	1.07–2.45	0.022	1.79	1.00–3.23	0.050
T3	2.50	1.57–3.99	<0.001	1.99	1.01–3.94	0.047
Tumor grade						
G1	Reference			Reference		
G2	1.23	0.74–2.05	0.425	1.15	0.55–2.41	0.703
G3	1.64	0.99–2.73	0.056	1.73	0.84–3.57	0.135
Node metastasis						
No	Reference			Reference		
Yes	7.46	5.09–10.95	<0.001	7.97	4.54–14.01	<0.001
ER						
Positive	Reference			Reference		
Negative	1.92	1.34–2.75	<0.001	1.89	1.13–3.15	0.015
PR						
Positive	Reference			Reference		
Negative	1.18	0.82–1.68	0.375	1.15	0.69–1.90	0.591
HER2						
Negative	Reference			Reference		
Positive	1.53	1.05–2.23	0.026	1.95	1.15–3.30	0.013
Casting-type calcification						
No	Reference			Reference		
Yes	1.65	1.07–2.56	0.024	1.95	1.08–3.53	0.026

HR, hazard ratio.

### Stratification by tumor size

Because most of previous studies only focused on small tumors, we investigated the effect of casting-type calcification on DFS and OS according to tumor size (Table 4). We adjusted the effects of other possible prognostic factors in a multivariate Cox proportional hazards regression model. The other factors were axillary lymph node status, tumor grade, PR, ER, and HER2. Casting-type calcification in T2 tumors was a significant prognostic factor both for OS and DFS (OS HR = 3.61; 95% CI, 1.43–9.10, *P* = 0.006; DFS HR = 2.33; 95% CI, 1.17–4.66, *P* = 0.016). Casting-type calcification in T3 tumors was significantly associated with OS (HR = 4.70; 95% CI, 1.31–16.93, *P* = 0.018). This trend only showed borderline significance for DFS (HR = 2.37; 95% CI, 0.98–5.37, *P* = 0.056). Casting-type calcification in T1 tumors was not significantly associated with either OS or DFS (OS HR = 0.77; 95% CI, 0.23–2.53, *P* = 0.666; DFS HR = 0.91; 95% CI, 0.40–2.10, *P* = 0.833).

**Table 4:** Influence of casting-type calcification on disease-free survival and overall survival in different tumor sizes.

Tumor size	Disease-free survival		Overall survival	
	Multivariate HR* (95% CI)	Multivariate *P*	Multivariate HR* (95% CI)	Multivariate *P*
T1 (n = 549)	0.91 (0.40–2.10)	0.833	0.77 (0.23–2.53)	0.666
T2 (n = 454)	2.33 (1.17–4.66)	0.016	3.61 (1.43–9.10)	0.006
T3 (n = 152)	2.37 (0.98–5.37)	0.056	4.70 (1.31–16.93)	0.018

*Adjusted for tumor grade, axillary node status, ER, PR, HER2.

### First sites of locoregional recurrence and distant metastases

In the casting group, there were 20 events of recurrence or distant metastases. However, in the non-casting group, there were 70 events (Table 5). The first sites of locoregional recurrence and distant metastases were also analyzed between the two groups. In the casting group, bone metastases were the most common metastases (10 cases), which was followed by locoregional recurrence (4 cases), lung metastases (2 cases), distant lymph node metastases (2 cases), liver metastases (1 case), and multiple metastases (1case). In the non-casting group, bone metastases were also the most common metastases (25 cases), followed by locoregional recurrence (21 cases), lung metastases (7 cases), distant lymph node metastases (6 cases), liver metastases (5 cases), multiple metastases (4 cases), and brain metastases (3 cases). However, the distributions of the first sites of locoregional recurrence and distant metastases between the two groups were not significantly different (*P* > 0.05).

**Table 5:** First sites of locoregional recurrence and distant metastases.

	Locoregional, No. (%)	Distant metastases, No. (%)					
		Multiple	Distant node	Bone	Lung	Liver	Brain
Casting group (n = 20)	4 (20.00)	1 (5.00)	2 (10.00)	10 (50.00)	2 (10.00)	1 (5.00)	0 (0.00)
Non-casting group (n = 70)	21 (30.00)	4 (5.71)	6 (8.57)	25 (35.71)	7 (10.00)	5 (7.14)	3 (2.86)
P*	0.379	0.902	0.843	0.248	1.000	0.735	0.346

*Two-tailed chi-squared test and likelihood-ratio chi-squared test. Differences were considered significant at *P* < 0.05.

## Discussion

Breast cancer is the most frequently occurring cancer in Chinese women. It has an age-standardized rate of 21.6 cases per 100,000 women^14^. Recently, mammographic imaging has become an important technique for the early detection and diagnosis of breast cancer in a number of countries, including China^15^. In addition to their usefulness for detection and diagnosis, several studies have also reported that mammographic casting-type calcifications may be indicative of the prognosis of a patient. Tabar *et al*. studied 343 patients with small, screen-detected breast tumors and they observed far higher mortality rates in patients with casting-type calcifications relative to those without casting-type calcifications^4^. Subsequently, Tabar *et al*. studied 714 patients with tumors that were less than 15 mm in size^16^. This study suggested that casting-type calcification was an unfavorable prognostic factor (9.19-fold increased mortality for patients with casting-type calcifications). Thurfjell *et al*. investigated 96 consecutive cases of invasive breast cancers that were 1–9 mm with or without mammographic calcification. They reported that casting-type calcification conferred a significantly higher risk of death independently, taking grade and node status into account^17^. Peacock *et al*. assessed 50 women diagnosed with small invasive tumors and associated casting-type calcifications with tumor size and lymph node involvement compared to matched controls^18^. This study showed that casting-type calcification was a significantly unfavorable prognostic factor for patients with small breast cancer. Tsau *et al*. found that casting-type calcifications were associated with a 3.47-fold increased hazard ratio for mortality in a study of 498 patients diagnosed with invasive breast cancer, after adjusting for other prognostic factors^5^. Similarly, Bennett *et al*. found, in young women with screening detected breast cancer, that mammographic comedo (casting) calcification had independent prognostic significance (HR 3.00, 95% CI 1.13–7.99).

No other studies, however, confirmed the prognostic value of casting-type calcifications. Mansson *et al*. reported that casting-type calcification was not a statistically significant prognostic factor in a study of 515 women with small breast cancers, although there was a trend toward decreased survival^7^. Evans *et al*. investigated a consecutive series of 470 women with screening-detected invasive breast cancers and found the presence or absence of mammographic comedo (casting) calcification did not have an influence on breast-cancer-specific survival^8^. James *et al*. showed the presence of casting-type calcifications was associated with small, high-grade tumors, but not with survival^6^. Another study recently reported that tumors with casting-type calcifications were associated with worse survival rates than tumors with non-casting type calcifications (*P* = 0.06)^11^. This trend, however, only showed borderline significance.

Many studies have also revealed links between casting-type calcifications and the risk for recurrence of breast cancer. A retrospective study of 55 high-risk breast cancer patients reported significant associations between the risk of relapse and the presence of casting calcifications (HR 3.048, 95% CI 1.116–8.323, *P* = 0.030)^19^. Qi *et al*. reported a significant increased rate of local recurrence in patients with mammographic calcifications, especially for linear (many of them were casting-type) or segmental distribution^20^. In a study of 937 cases of invasive breast cancers, the association between microcalcification and recurrence was not significant^21^.

The previous studies had some limitations. First, most previous studies only assessed small breast tumors and most of the tumors were detected by mammography screening. Secondly, the sample size of casting-type cohort of most previous studies was small. Among previous studies, the largest casting-type cohort was only of 119 cases^11^. The small sample size would inevitably limit the effect of survival analysis and multivariate analysis. Thirdly, most of the earlier studies lacked immunohistologic information, and the treatment methods were not similar to those of recent years. For example, in the time of early studies, trastuzumab or aromatase inhibitor were not available, which limited the conclusion to be applied in modern clinical practices.

Our study was the first to focus on the prognostic value of casting-type calcification in Chinese breast cancer patients, and its casting-type cohort was the largest up to now. This study included both screening-detected small tumors and more advanced ones, and had complete clinical and pathologic information, which facilitated multivariate and subgroup analysis. The results of our study showed that the patients exhibiting mammographic casting-type calcifications had significantly worse OS and DFS, and this result remained even after adjusting other prognostic factors in multivariate analysis. However, in subgroup analysis of tumor size, we found the prognostic strength was more influential in T2 or T3 tumors, but not so significant in T1 tumors. This finding is contrary to some earlier studies focusing on small tumors. First, this discrepancy may be related to the sampling of the surgical specimens. In earlier studies, sampling of ductal carcinoma *in situ* (DCIS) to look for invasive areas was not as thorough as is performed currently. It is possible that additional small invasive foci of breast cancer might go undiagnosed, which would result in a larger true invasive tumor size than initially reported^6^. Secondly, in the days of some early studies, trastuzumab, aromatase inhibitor and a lot of other chemotherapy drugs were not available. The prognostic potential of casting-type calcifications demonstrated in earlier studies were not confirmed in later studies, including our study, because the primary survival difference may not be significant with the use of very effective adjuvant treatment nowadays.

Casting-type calcifications having a significant association with other prognostic factors, including tumor size, node status, grade, and biomarkers, has also been reported. Tabar *et al*. showed that casting-type calcifications were significantly associated with a poorer histologic grade (OR 7.04) and a positive lymph node status (OR 3.29). Ling *et al*. stated that patients with malignant calcification (including casting-type) had larger tumor sizes and more lymph node involvement; however, the tendency toward a higher grade was not significant. Many other studies have also indicated an ER/PR negativity rate and an increased rate of HER2 overexpression in patients with casting-type calcifications^5,7,19^. A recently reported microarray analysis showed the *ERBB2* gene had greater expression in patients with highly suspicious calcifications^10^. However, another recent study showed a significant positive association between breast osteoblast-like cells(BOLCs) and ER expression rather than HER2 expression. And BOLCs were vital to the production of casting-type calcification^22^. The present study showed that casting-type calcification was more common in patients with axillary node involvement, ER/PR negativity and HER2 overexpression, which is in agreement with most previous studies. In order to adjust the prognostic effect of these factors, we performed a multivariate analysis that showed casting-type calcification was an independent prognostic factor for both DFS and OS.

The mechanism for casting-type calcification conferring a poor prognostic effect is not understood. Tabar *et al*. proposed that casting-type calcification was strongly linked to “neoductgenesis,” which might lead to extensive lymphatic and hematogenous spread of cancer cells^16,23,24^. Morgan *et al*. reported that calcifications of breast cancer cell lines could stimulate mitogenesis through synthetic hydroxyapatite (HA) particles^25–27^. Cox *et al*. showed that formation of mammary HA particles was a process that was cell-specific regulated, which creates an osteomimetic niche that potentially enhances breast tumor progression^28^. Cooke *et al*. presented evidence that calcium HA crystals exerted significant biological effects on the surrounding cells, and they played an important role in amplifying the pathological processes of breast cancer^29^. Another study indicated that microcalcifications in breast cancer could be affected by the epithelial–mesenchymal transition (EMT), which was a common phenomenon in malignant lesions of breast^30^. Sharma *et al*. reported that bone morphogenetic proteins (BMPs) and tumor associated macrophages (TAMs) were strongly associated with microcalcifications of invasive breast cancer cells^31^. Another study reported some osteoclastogenic factors which were important to calcification formation of breast cells^32^. In addition, HA was important to mediate the expression of cyclooxygenase-2 (COX2), which exerts multiple tumor-promoting effects in breast cancer cells^25,33,34^. Nevertheless, these studies focused on all kinds of calcifications, rather than solely on casting-type calcification. A recent report suggested that casting-type calcifications were mostly made of hydroxyapatite magnesium substitutions and were associated with the breast cancer subtypes that had the poorest prognosis. Breast cancer cells near the microcalcifications also expressed higher levels of bone mineralization factors, which might lead to tumorigenic process^35^.

There are some limitations to this study. First, retrospective studies should be interpreted with caution because of the potential for confounding factors. Second, this study was carried out at a single center and therefore the prognostic impact of casting-type calcification should be validated by studies performed at other institutions. Third, the mammogram results were reviewed by one radiologist; therefore, the reproducibility of the results regarding the presence or absence of casting-type calcification was not addressed.

In conclusion, our findings revealed that casting-type calcification was more common in patients with axillary node metastasis, ER-negativity and HER2 overexpression. The OS and DFS were significantly worse in patients with casting-type calcification, even after adjusting other prognostic factors in multivariate analysis. The prognostic value was more significant in T2 or T3 tumors than small tumors. Further investigation is needed to clarify the mechanism for casting-type calcification having an effect on breast cancer prognoses.

## Methods

### Ethics statement

This study was approved by the independent “ethical committee/institutional review board of Peking Union Medical College Hospital”. The committee waived the need for written informed consent because it was a retrospective study. We obtained permission of PUMCH to collect data from the Breast Surgery Department Database. Our study was carried out in accordance with the relevant guidelines and regulations.

### Patients

Data was collected for patients diagnosed with invasive breast cancers in PUMCH between January 2010 and January 2013. Patients were required to have received pre-surgery mammograms and definitive surgery of both the breast and axilla. The exclusion criteria were a previous history of other malignant neoplasms, including distant metastasis at diagnosis, breast cancer, neoadjuvant therapy received prior to surgery, bilateral breast cancer at diagnosis, and T4 tumors according to the American Joint Committee on Cancer (AJCC) TNM Staging System (in our center, about 38% of T4 patients did not have pre-surgery mammographic examination because of skin involvement or other reasons, therefore, this research excluded T4 tumors to avoid bias).

All the eligible patients had adequate medical information and were followed up. The last follow-up date was January 19, 2018.

### Data collection

We collected data on patients’ tumor characteristics and demographic information from medical records, including age, menstrual status, tumor grade, tumor size, axillary lymph node status, PR, ER, HER2, and treatment methods. Follow-up data were reviewed from the follow-up system at the center. The patients treated at PUMCH were all encouraged to appear for follow up visits after surgery. Follow up by phone was carried out if a patient did not show up for their appointment.

The details of fluorescence *in situ* hybridization (FISH) analysis and immunohistochemical (IHC) analysis are: Vendor (Ventana Benchmark XT, Tucson, AZ, USA), Detection (iView/DAB; Ventana), and Clone (ER: SP1, pre-diluted; PR: 1R2, pre-diluted; HER2: 4B5, pre-diluted). HER2 FISH analysis was carried out using the Her-2 probe kit (Abbott Molecular, USA) according to the manufacturer’s instructions.

Mammograms were acquired with dedicated mammography units (Senograph 2000D, General Electric Medical Systems, Milwaukee). Mammograms were performed with at least two views per breast and they were medio-lateral oblique and cranio-caudal views. Additional views or spot compression views were obtained when appropriate.

**Figure 3:**
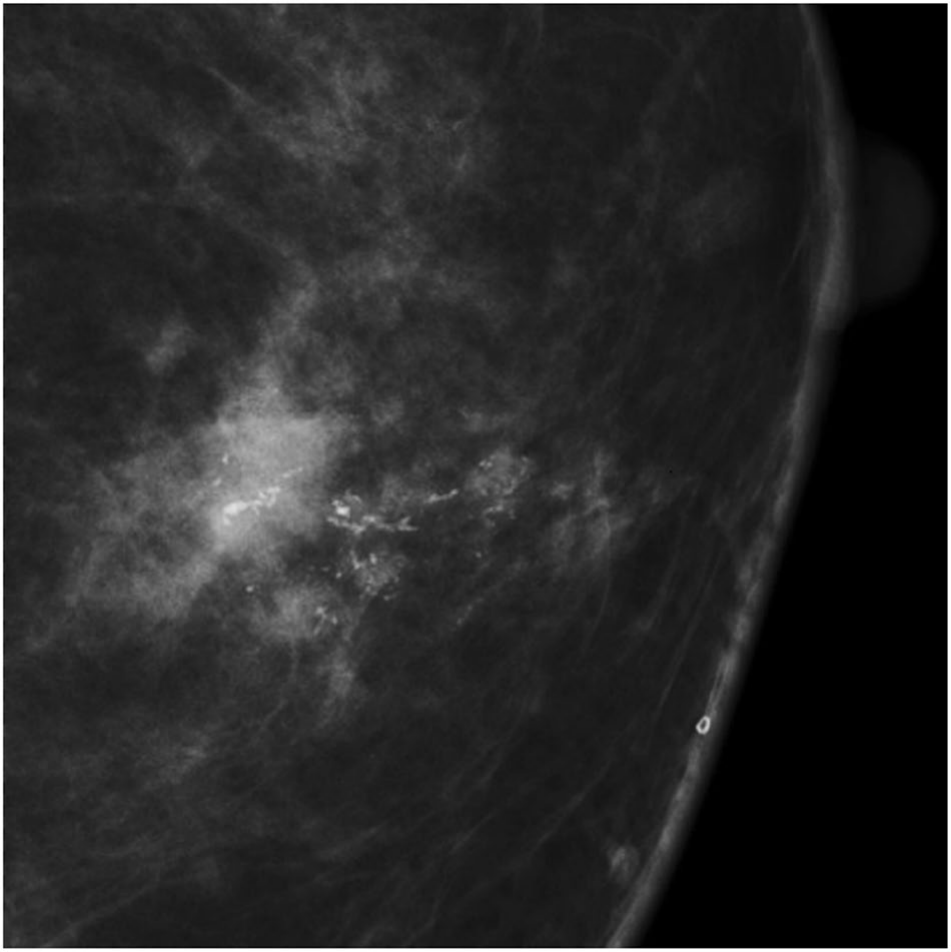
Typical casting-type calcifications seen on mammography.

Casting-type calcification was defined as a long, fine linear branching structure on mammography (Fig. 3). An experienced radiologist (J Cao) reviewed all the images to determine the presence or absence of casting-type calcifications. This was done blinded to the survival status of the patient and other medical information. The patients were divided into two groups accordingly: the casting group (with casting-type calcification) and the non-casting group (without calcification or with non-casting type calcifications, e.g., egg-shell calcification, rod-shaped calcification, popcorn calcification, powdery calcification, granular-type calcification and other calcifications).

The endpoint of this study was OS and DFS. OS was calculated from the initiation of treatment to death. DFS was calculated from the initiation of treatment to second primary cancer, recurrence, or death without evidence of second primary cancer or recurrence. Breast cancer recurrence included loco-regional recurrence and distant metastases.

### Statistical analysis

Quantitative data were analyzed with a Student’s t-test. Categorical data were assessed with a multivariate logistic regression model and two-sided chi-squared test. DFS and OS were estimated with the Kaplan-Meier method. Two-sided log-rank tests were used for time-to-event endpoints. Multivariate survival analysis with adjusted pathological factors that were known to affect patient survival, including lymph node status, tumor size, histologic grade, PR, ER, and HER2, was performed with a multivariate Cox proportional hazards regression model. Significance was set at P < 0.05. Statistical analyses were performed with the STATA statistical software package (version 14.0, Texas, USA).

## Data Availability

All data that were generated or analyzed in this study are included in this article.
